# Designing Cell-Type-Specific Promoter Sequences Using Conservative Model-Based Optimization

**DOI:** 10.1101/2024.06.23.600232

**Published:** 2024-06-23

**Authors:** Aniketh Janardhan Reddy, Xinyang Geng, Michael H. Herschl, Sathvik Kolli, Aviral Kumar, Patrick D. Hsu, Sergey Levine, Nilah M. Ioannidis

**Affiliations:** 1 University of California, Berkeley

## Abstract

Gene therapies have the potential to treat disease by delivering therapeutic genetic cargo to disease-associated cells. One limitation to their widespread use is the lack of short regulatory sequences, or promoters, that differentially induce the expression of delivered genetic cargo in target cells, minimizing side effects in other cell types. Such cell-type-specific promoters are difficult to discover using existing methods, requiring either manual curation or access to large datasets of promoter-driven expression from both targeted and untargeted cells. Model-based optimization (MBO) has emerged as an effective method to design biological sequences in an automated manner, and has recently been used in promoter design methods. However, these methods have only been tested using large training datasets that are expensive to collect, and focus on designing promoters for markedly different cell types, overlooking the complexities associated with designing promoters for closely related cell types that share similar regulatory features. Therefore, we introduce a comprehensive framework for utilizing MBO to design promoters in a data-efficient manner, with an emphasis on discovering promoters for similar cell types. We use conservative objective models (COMs) for MBO and highlight practical considerations such as best practices for improving sequence diversity, getting estimates of model uncertainty, and choosing the optimal set of sequences for experimental validation. Using three relatively similar blood cancer cell lines (Jurkat, K562, and THP1), we show that our approach discovers many novel cell-type-specific promoters after experimentally validating the designed sequences. For K562 cells, in particular, we discover a promoter that has 75.85% higher cell-type-specificity than the best promoter from the initial dataset used to train our models.

## 1. Introduction

Gene therapies treat diseases through the delivery and expression of therapeutic genetic cargo in disease-associated cells and tissues. Expression is controlled by a promoter sequence, a short regulatory DNA sequence (typically up to a few hundred base pairs (bp) long) placed upstream of the coding region. An ideal promoter for gene therapy would differentially induce expression in targeted cells while repressing expression in all other cells to increase effectiveness and reduce side-effects; i.e. it should be cell-type-specific and induce differential expression. However, although there are over 400 types of cells in the human body (Tabula Sapiens Consortium, 2022), very few cell-type-specific promoters are known.

Traditional methods to design cell-type-specific promoters rely heavily on manual curation or involve tiling known cis-regulatory elements (CREs) or transcription factor (TF) binding motifs ((Miao et al., 2000; Selvakumaran et al., 2001; Yun et al., 2008; Nissim et al., 2017; Wu et al., 2019) among others). These methods are difficult to automate and are not guaranteed to work, especially in less studied cell types. Directed evolution can be used to automate promoter design but is expensive to perform, typically requiring many experimental validation steps to continually improve designs, and does not make the best use of available data. offline model-based optimization (MBO) has recently been used to design promoters and other CREs in a data-driven manner (Linder et al. (2020); Wang et al. (2020); Jores et al. (2021); LaFleur et al. (2022); Gosai et al. (2023) among others). The offline MBO approach involves building machine learning (ML) models of promoter-driven expression (PE) using experimental measurements and then optimizing the promoter sequence using model predictions as surrogates for experimental measurements. offline MBO has the potential to accelerate promoter discovery by being automated and relatively data-efficient; however, the field is lacking a generalizable MBO-based framework for designing cell-type-specific promoters in a data-efficient manner, while systematically accounting for practical considerations including:

1. **Data-efficiency:** Measuring PE is expensive and time-consuming, necessitating the effective use of available data during design. Most previous work on offline MBO for promoter design has used large PE datasets for model training (e.g. from massively parallel reporter assays, MPRAs) that are only available for a few well-studied cell types, and their modelling strategies have not been evaluated when designing cell-type-specific promoters for less-studied cell types using small PE datasets.
2. **Minimizing adversarial designs:** Using models for optimization, rather than just prediction, presents key additional challenges, since naïvely optimizing designs based on predicted differential expression (DE) can lead to *adversarial* designs that fool a model into outputting desirable values while having undesirable values in reality. Previous work using optimization techniques such as *in silico* directed evolution, gradient ascent, or generative models do not account for such designs.
3. **Selecting design candidates while accounting for sequence diversity and uncertainty in estimates of a sequence’s goodness:** Offline MBO can design many sequences that are predicted to be cell-type-specific by the PE model. However, due to limited budgets and time for wetlab experiments, typically only a subset of the candidates can be validated. Thus, we need a systematic approach to choose promising candidates from a larger set of designs in a principled manner. During selection, it is important to consider both the diversity of the chosen candidates and the uncertainty in model predictions. Choosing diverse candidate designs is important (1) to improve the chances of successfully discovering a cell-type-specific promoter upon experimental validation, since we lack complete information about how a sequence controls PE, and (2) to explore sequence space more effectively during several rounds of design, leading to progressively better models and designs. It is also important to choose candidates that have low uncertainty in model predictions while also balancing their potential goodness. This sequence selection process is crucial for a principled MBO workflow and has been overlooked by previous work.

Here we present a systematic framework and case study for applying offline MBO to the real world problem of promoter design, backed by empirical evidence from wetlab experiments evaluating differential expression in three closely related blood-based cell types. To improve data-efficiency through transfer learning, we follow previous work (Reddy et al., 2024) and pretrain sequence-based deep learning (DL) models of PE using large existing MPRA datasets that measure PE in different contexts, and fine-tune them using a small PE dataset collected from the target cell types. To address adversarial designs, we extend the conservative objective models (COMs) framework (Trabucco et al., 2021) to our setting: while fine-tuning models, we use a loss term that reduces the predicted DE of adversarial designs. These fine-tuned models are then used to design cell-type-specific promoters by performing gradient ascent on the inputs. Upon generating multiple candidate sequences, we propose a selection scheme to choose a diverse yet effective subset of final candidates that uses uncertainty estimates from an independently trained ensemble. We use our workflow to design cell-type-specific promoters for three blood cancer cell lines (Jurkat, K562, and THP1) and present experimental evidence of its effectiveness. Existing studies have designed promoters for markedly different cell types derived from different tissues (Gosai et al., 2023), a simpler problem since such cell types typically have very different regulatory environments with different transcription factors (TFs) expressed. We show that our MBO approach works in a more difficult setting where we aim to design promoters that are only expressed in one of three highly similar blood-based cell lines. Our designs improve upon the cell-type-specificity of sequences from the fine-tuning dataset, **improving 72.12% of sequences in Jurkat cells and 80.48% in K562 cells**. Additionally, **in K562 cells, we identify a promoter with 75.85% higher DE than the best promoter** from the fine-tuning dataset. These results suggest that applying our approach over multiple rounds of modelling, design, and experimental validation will enable the discovery of promoters with even higher cell-type-specificity, by shifting the training distribution towards higher DE sequences at each iteration.

## 2. Related work

### 2.1. Promoter design methods

Promoters have traditionally been designed using heuristics and manual curation. For example, Selvakumaran et al. (2001) and Yun et al. (2008) found cell-type-specific promoters for ovarian and breast cancer cells, respectively, by identifying genes that are differentially expressed in these cells and showing that their promoters exhibit cell-type-specificity. Nissim et al. (2017) designed cell-type-specific promoters for ovarian and breast cancer cells by identifying differentially expressed TFs and tiling their binding motifs. However, these studies do not provide a generalizable method to design cell-type-specific promoters for any given cell type. In an attempt to address this issue, recent work has sought to develop more automated and data-driven design methods. Kotopka and Smolke (2020) and Jores et al. (2021) used sequence-based convolutional neural network (CNN) models alongside simple optimization techniques such as *in silico* directed evolution and gradient ascent to design promoters for high expression in yeast and various plant cells, respectively. Wang et al. (2020) generated E. coli promoters by training a generative adversarial network (GAN) on natural E. coli promoters and then using a PE predictor to further filter generated sequences. Gosai et al. (2023) trained a sequence-based CNN model of PE using a large MPRA dataset from three very different cell lines. They used this model with three design algorithms to produce cell-type-specific promoters that they show to be effective and diverse. Here, we build on previous work in three crucial ways. First, we exploit the benefits of pretraining on large existing PE datasets to build accurate models for target cell types in a more data efficient manner compared to previous methods. Second, we leverage COMs (Trabucco et al., 2021), a powerful offline MBO method, to design promoters using these models more effectively, minimizing adversarial designs. Finally, we propose a sequence selection strategy that explicitly accounts for sequence diversity and model uncertainty to choose a subset of sequences for experimental validation from a larger set of design candidates.

### 2.2. Offline MBO for designing biological sequences:

Apart from COMs, other offline MBO methods have been proposed for designing biological sequences. While COMs only requires discriminative models, most of the other methods rely on generative models. For example, Brookes et al. (2019) built variational autoencoders (VAEs) that generate desirable designs by iteratively improving the VAE and its designs, guided by a design model. Jain et al. (2022) used GFlowNets for sequence design, another class of generative models that are trained to output designs that span all modes of some design model’s prediction distribution. Linder et al. (2020)’s deep exploration networks (DENs) are generative models that are trained to output diverse yet desirable sequences and again use design model predictions to guide the training process. They experimentally validated their method and showed that alternative polyadenylation (APA) sites designed using DENs were better than those designed using gradient ascent. Here we use DENs as a baseline in our analyses, since it was validated using wetlab experiments.

## 3. Background and motivation

### 3.1. The cell-type-specific promoter design problem

A typical promoter design experiment ((Schlabach et al., 2010; Kotopka and Smolke, 2020; Jores et al., 2021; LaFleur et al., 2022) among others) starts by identifying target cell types and defining an objective such as increasing the absolute or differential expression levels of some gene. Then, an initial set of candidate promoters is prepared by leveraging existing data sources and heuristics, or even using random sequences. To test the efficacy of these promoters, reporter assays that collect PE measurements for a large batch of promoters simultaneously are used (batch sizes range from a few hundred to many thousands or even millions). Then, using these initial PE measurements, better promoters can be designed using various methods such as directed evolution, shuffiing motifs enriched in desirable sequences, or offline MBO. The designed promoters then need to be experimentally validated using another reporter assay. If a sufficiently desirable promoter is not discovered, the design and experimental validation steps can be repeated.

Here we focus on designing short cell-type-specific promoters in a data-constrained setting. In this setting, we have a set of target cell types C, from which we have a small PE dataset – a few thousand sequences and corresponding PE measurements from all cell types in C. Gene delivery mechanisms impose constraints on the length of DNA that can be delivered. Thus, we can often only accommodate promoters that are at most a few hundred base pairs (bp) long. For any target cell type tc∈C, we define the DE of a promoter x (i.e. its cell-type-specificity) as:

(1) $${\mathrm{DE}}^{tc}\left(x\right)=\frac{1}{\left(\left|C\right|-1\right)}\sum_{oc\neq tc}\left[{\mathrm{PE}}^{tc}\left(x\right)-{\mathrm{PE}}^{oc}\left(x\right)\right]$$

where ${\mathrm{PE}}^{c}\left(x\right)$, c∈C, is the experimentally measured expression value induced by x in cell type c. Our goal is to design sequences that maximize ${\mathrm{DE}}^{tc}$ - the average difference in PE between the target cell type tc and the other cell types.

### 3.2. Offline MBO

An offline MBO algorithm aims to produce designs that maximize some objective function, using a provided static dataset $𝒟=\{(x_{i},y_{i})\}$ of designs $x_{i}$ and a corresponding measurement of the objective value $y_{i}$. The algorithm analyzes this dataset and produces an optimized candidate design $x^{*}$, which is evaluated against the true objective function. This process often involves learning a proxy objective $f_{\theta }$ mapping designs to corresponding objective values, which we hereafter refer to as the **design model**. Optimization is then performed to find an input which maximizes this learned design model: $x^{*}=\arg\max_{x}f_{\theta }\left(x\right)$, using methods such as *in silico* directed evolution, gradient ascent, or by building generative models.

As many offline MBO algorithms are data-efficient, they are suitable for promoter design in data-constrained settings. When several rounds of promoter design are performed, we can use all available PE measurements from previous rounds as the “static” dataset for offline MBO. In our workflow, we propose an additional selection step after running offline MBO that diversifies the final designs if needed, making the pipeline more suitable for multi-round optimization.

## 4. Our MBO workflow for designing promoters in data-constrained settings

**Figure 1:**
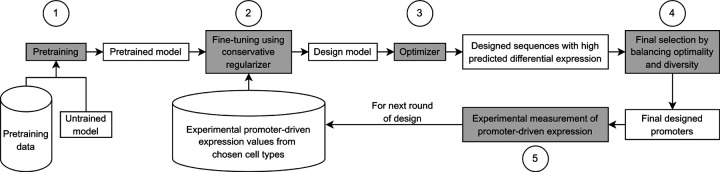
Our workflow for designing cell-type-specific promoters. Five main steps are highlighted in grey: (1) pretrain a base model using existing large genomic datasets; (2) fine-tune the pretrained model using the experimentally measured PE data collected so far, while also using a conservative regularizer; (3) use a gradient ascent-based optimizer to design sequences that have high predicted DE; (4) apply a final sequence selection algorithm that balances optimality and diversity to choose a smaller subset of the designed sequences; (5) experimentally measure the PE of the selected designed sequences. The last four steps can be repeated when running multiple rounds of design iterations.

In this section, we present our approach to design cell-type-specific promoters that accounts for key practical considerations. Figure 1 summarizes the workflow.

### 4.1. Building models of promoter-driven expression in a data-efficient manner

As previously mentioned, offline MBO algorithms need a design model that approximates the objective function. When designing cell-type-specific promoters for some target cell type tc∈C, our objective function is ${\mathrm{DE}}^{tc}\left(x\right)$ that is in turn computed using all ${\mathrm{PE}}^{c}\left(x\right)$, c∈C, values. Therefore, instead of directly modelling ${\mathrm{DE}}^{tc}\left(x\right)$, we simultaneously model all ${\mathrm{PE}}^{c}\left(x\right)$ values using multi-task learning (MTL). This allows us to use one design model to design cell-type-specific promoters for all c∈C. In this section, we provide guidelines to build these models based on the findings of Reddy et al. (2024), which studies model architectures and transfer learning strategies for modelling PE.

#### Model architecture

Recent work has shown that genomic data can be accurately modelled using sequence-based models – models that take in one-hot encoded DNA sequences as input to predict associated experimental assay measurements such as gene expression, histone modifications, and TF-binding (e.g. (Zhou and Troyanskaya, 2015; Agarwal and Shendure, 2020; Avsec et al., 2021)). Reddy et al. (2024) find that a model consisting of convolutional layers followed by transformer layers (MTLucifer) outperforms other architectures when used to model PE without any transfer learning.

#### Transfer learning to boost accuracy

Effective offline MBO is dependent on accurate design models, and building accurate models often requires large datasets. However, collecting a large dataset with experimental measurements of PE in multiple cell types is expensive and time-consuming. In such data-constrained settings, transfer learning can be very beneficial. Indeed, Reddy et al. (2024) showed that pretraining a sequence-based model on large related genomic datasets before fine-tuning it on a smaller PE dataset from target cells leads to significantly better modelling of the smaller dataset compared to training exclusively on the smaller dataset. The three best approaches that they identified are:

1. **Linear probing of Enformer** (Avsec et al., 2021) **predictions using Lasso** (Tibshirani, 1996): Enformer is a large sequence-based model (again, consisting of convolutional and transformer layers) trained on thousands of epigenomic datasets from humans and mice, allowing it to accurately learn sequence features that control gene expression. Since it is expensive to train Enformer (3 days on 64 TPU v3 cores), we often need to rely on the pretrained models published by Avsec et al. (2021), limiting flexibility in terms of changing architectures or using alternate deep learning (DL) libraries. It can also be difficult to use Lasso with gradient-based optimization techniques when performing offline MBO. Therfore, this approach is only recommended when the published pretrained Enformer model can be easily incorporated into your code base without any architectural changes, and when the offline MBO algorithm you are using is not reliant on gradients (e.g. CbAS (Brookes et al., 2019)).
2. **Fine-tuning Enformer by using its embed-dings to predict PE in target cells:** The performance of this approach is very similar to the previous approach. Moreover, using fine-tuning instead of Lasso allows us to easily employ gradient-based offline MBO methods. Thus, this approach is recommended when the pretrained Enformer model is compatible with your code base and you do not wish to make any changes to its architecture that would necessitate re-training.
3. **Pretraining MTLucifer on large existing MPRA datasets before fine-tuning it to predict PE in target cells:** Certain MPRA datasets (e.g. (Ernst et al., 2016; van Arensbergen et al., 2019)) measure PE in selected cell lines from a large number of sequences. These data are useful for transfer learning, since models can learn a broad set of features that are generally useful for predicting PE. This approach is very inexpensive (33 hours for pretraining on a single Nvidia A40 GPU), which makes it easier to experiment with architectures and hyperparameters. Hence, this approach is recommended when you want to use custom architectures for modelling your data, or if you want flexibility in the DL libraries used to build your code base.

Using any of these approaches should yield an accurate model of PE in our target cells. However, since we focus on performing offline MBO using COMs, which require differentiable design models, only the last two approaches are compatible with our workflow.

### 4.2. Performing offline MBO while mitigating adversarial designs using COMs

Once we have accurate design models, we can couple them with offline MBO algorithms to design promoters. However, during the design process, we need to address adversarial designs that might arise due to the distribution shift problem. We describe the problem below and how it can be mitigated using COMs (Trabucco et al., 2021). Then, we specify how COMs can be used to design cell-type-specific promoters.

#### Distribution shift problem in offline MBO and conservative regularization

While we can train an accurate design model $f_{\theta }\left(x\right)$, it may still suffer from generalization failures common to supervised regression models, especially when designed sequences are far away from the training data’s distribution i.e. when there is a distribution shift. Such adversarial sequences can be easily designed by optimizers by heavily mutating training sequences, especially those that are already good. To alleviate this issue, we suggest learning and using *conservative* models of the objective function, or COMs (Trabucco et al., 2021), as design models. These models are trained using a *conservative regularizer* that penalizes high predictions on the set of unseen and potentially undesirable promoters $\mu \left(x\right)$ which appear promising under the current design model, thus preventing the optimizer from designing adversarial promoters. Concretely, conservative models use the following loss:

(2) $$\underset{\theta }{\min}\underset{\text{:=}\mathrm{supervised}\text{loss}}{\underset{︸}{E_{x∼𝒟}\left[{\left(f_{\theta }\left(x\right)-y\right)}^{2}\right]}}+\alpha \underset{\text{:=}\mathrm{conservative}\text{regularizer}}{\underset{︸}{E_{x∼\mu }\left[f_{\theta }\left(x\right)\right]-E_{x∼𝒟}\left[f_{\theta }\left(x\right)\right]}}$$

where α **is the conservatism coefficient** and controls the degree of “conservatism”.

#### Training design models for designing cell-type-specific promoters using conservative regularization

The previous subsection mentions two approaches for building differentiable design models. In either approach, we have a pretrained model (either a pretrained Enformer or MTLucifer model) that is fine-tuned using a small dataset from the target cells. To incorporate conservative regularization into the fine-tuning process, we modify the loss. Let us denote the model prediction for PE in cell type c as ${\mathrm{PE}}_{\theta }^{c}\left(x\right)$, and the predicted DE in c as ${\mathrm{DE}}_{\theta }^{c}\left(x\right)$, then the overall fine-tuning objective is:

(3) $$\underset{\theta }{\min}\sum_{c\in C}\left\{E_{x∼𝒟}\left[{\left({\mathrm{PE}}_{\theta }^{c}\left(x\right)-{\mathrm{PE}}^{c}\left(x\right)\right)}^{2}\right]+\alpha \left(E_{x∼\mu_{c}}\left[{\mathrm{DE}}_{\theta }^{c}\left(x\right)\right]-E_{x∼𝒟}\left[{\mathrm{DE}}_{\theta }^{c}\left(x\right)\right]\right)\right\}$$

In contrast to the canonical formulation of COMs in Eqn 2, we use DE in the regularization term, a value derived from the model’s expression predictions, instead of using expression itself, as our goal is to maximize DE and we want to avoid adversarial sequences with erroneously high predicted DE using the conservative regularizer. Furthermore, since we use a single design model for all target cells, the regularization term needs to be computed for all target cells. In contrast, the canonical COMs formulation works for only one objective function. Similar to Trabucco et al. (2021), to get distribution $\mu_{c}\left(x\right)$ consisting of sequences with potentially overestimated DE in c in every training step, we can use the Adam optimizer (Kingma and Ba, 2015) to perform T steps of gradient ascent on ${\mathrm{DE}}_{\theta }^{c}\left(x\right)$ starting from sequences in the training batch (in our experiments: learning rate=0.5, $\beta_{1}=0.9$, $\beta_{2}=0.999$, and T=100). Since the DNA sequences are discrete, we perform gradient ascent in the probability simplex in the one-hot encoded space, parameterized by a softmax function. At the end of T steps of gradient ascent, we perform a hard clipping so that the resulting sequence is a valid one-hot encoding. The full fine-tuning objective is optimized till convergence and the model checkpoint with the lowest validation set loss is used as the design model. *Importantly, it is difficult to choose an α a priori. Therefore, we train multiple design models with different α values and use all of them to design candidate promoters (in our experiments, $\alpha \in \left\{0,0.0003,0.001,0.003,0.01,0.03\right\}$).*

#### Designing promoters using gradient ascent:

After training a design model, starting from each promoter in the fine-tuning dataset (henceforth referred to as the **starting sequence**), we apply the same gradient ascent-based optimization process as that used to build $\mu_{c}\left(x\right)$ to generate a design. This generation is performed using every design model (trained using different α values), yielding a large pool of candidate designed sequences.

### 4.3. Balancing diversity, optimality, and uncertainty for final sequence selection

From the design process described in the previous subsection, we get a large pool of candidate cell-type-specific promoters. Unlike typical offline MBO problems where an algorithm must produce only a single design for evaluation, as discussed in Section 3, designed promoters are typically evaluated in batches (say, of size K). Moreover, multiple iterations of promoter design can be performed. In this section we describe an algorithm to choose K promoters for experimental validation from the large set of candidates for cell type c, accounting for these facets of the promoter design problem.

Given that we have multiple design models, we cannot simply take the top K designs with highest predicted DE, since the predictions come from different models, making them difficult to compare with each other. Taking the top designs might also yield sequences that are very similar to each other. Finally, we also want to account for model uncertainty during selection and ideally choose high performing sequences that have low uncertainty.

#### Using an ensemble to get pessimistic estimates of DE that account for model uncertainty

To uniformly evaluate all candidate designed sequences, and potentially get more accurate predictions, we build an ensemble model consisting of models with slightly different architectures, using a train-validation-test split of the fine-tuning data that is different from the one used to build the design models. The constituent models are still pretrained prior to fine-tuning, but the conservative regularizer is not used during fine-tuning since these models are not directly used for design. To reduce the computational overhead for building the ensemble, one can use the same pretrained backbone in all constituent models but use different kinds of fine-tuning layers in each model. In our experiments, we use 36 models in the ensemble. To account for model uncertainty, we use the ensemble to compute ${\hat{\mathrm{DE}}}_{\gamma }^{c}\left(x\right)$, a pessimistic estimate of the DE in cell type c, for all candidates. The pessimistic estimate is defined as the lower confidence bound of the constituent models’ predictions and is computed as the mean minus the standard deviation of the predictions. *Using this pessimistic estimate in the final selection process allows us to choose sequences that most models are confident about, further reducing the risk of choosing adversarial designs.*

#### Final selection algorithm

Our ultimate goal is to maximize the expected efficacy of the designs in the set of final sequences selected for experimental validation. Intuitively, this can be achieved by: **(i)** ensuring that our designs have high predicted DE, and **(ii)** ensuring that the designs are as diverse as possible. Diversity is important as designs predicted to be optimal might not actually be optimal when they are experimentally validated. Having a diverse set of sequences increases the likelihood of *some* design being optimal, since we cover a broad region of the sequence space. We define a sequence set 𝒮’s diversity as:

$$D\left(𝒮\right)=\frac{1}{2}\sum_{x\in 𝒮,x^{\prime }\in 𝒮}\left[ℋ\left(x,x^{\prime }\right)+𝒦\left(x,x^{\prime }\right)\right]$$

where ℋ is the normalized Hamming distance between two sequences (ensuring overall diversity) and 𝒦 is the normalized Euclidean distance between the 6-mer frequency vectors of two sequences (ensuring diversity in potential TF-binding motifs composition). The distances are normalized by dividing them by their maximum possible values. To instantiate this intuition into a concrete strategy, we aim to select a set of K sequences ${𝒮}^{*}=\left\{x_{1}^{*},x_{2}^{*},\cdots ,x_{K}^{*}\right\}$ such that ${𝒮}^{*}$ is the optimal solution to the following optimization problem:

(4) $${𝒮}^{*}:=\arg\underset{𝒮}{\max}\sum_{x\in 𝒮}{\hat{\mathrm{DE}}}_{\gamma }^{c}\left(x\right)+\beta D\left(𝒮\right)$$

where ${\hat{\mathrm{DE}}}_{\gamma }^{c}\left(x\right)$ denotes a pessimistic estimate of the DE computed using an ensemble, and β **is the diversity coefficient** that controls the fitness vs. diversity trade-off. While in theory we can obtain ${𝒮}^{*}$ by optimizing over all sequences, doing so is computationally intractable. So, the optimization in Eqn 4 is performed using a greedy algorithm over a large subset of candidates from the previous step. The subset consists of candidates that have positive ensemble average predicted DE, and also have high predicted expression in the target cell type c ^1^ since this is also crucial for gene therapy applications. The algorithm sequentially chooses K sequences - in each step, given a set of sequences ${𝒮}^{\prime }$ already chosen to be in the final set, it computes ${\hat{\mathrm{DE}}}_{\gamma }^{i}\left(x\right)+\beta D\left({𝒮}^{\prime }\cup \left\{x\right\}\right)$ for every candidate sequence x not in ${𝒮}^{\prime }$, and chooses the sequence that maximizes this value for inclusion in the final set.

### 4.4. Making adjustments to the design process based on performance and diversity metrics

Here we discuss common problems that may arise during design and provide heuristics to address them.

#### Designed sequences are generally not predicted to have higher DE than the starting sequences from the fine-tuning dataset

This issue might arise when the conservative regularizer is too powerful, making it difficult for the optimizer to design good sequences. Here, reducing the conservatism coefficient α should help. However, there might also be problems with the dataset that hinder the optimizer. For example, if the fine-tuning dataset has very few sequences that drive some level of DE in the target cell type, the design models might be poor at modelling such sequences. To determine if this is the case, one can look at the distribution of DE values in the training set and if there are too few sequences that have positive DE in the target cell type (e.g. less than 25% of the dataset), the design problem might be too difficult to solve using the available data.

#### Designed sequences are not diverse

The diversity of a set of designs can be measured using multiple metrics. We focus on base pair entropy and average pairwise edit distance (can be any edit distance e.g. Hamming distance) as they are easy to interpret. If we are designing sequences of length L, base pair entropy can be computed at every position in the sequence by determining the frequency of the 4 DNA bases across all designed sequences. For a diverse sequence set, this metric should be close to 2 at every position (near uniform usage of the 4 bases). The average pairwise edit distance should be comparable to L (e.g. L/2). If these metrics are low, the diversity coefficient β can be increased to boost diversity. Using a more diverse set of starting sequences during optimization with gradient ascent can also improve the diversity of the designed sequences since we can converge upon different optima of the objective landscape.

## 5. Implementation and experimental evaluation of our approach

Next, we use the workflow proposed in the previous section to design 250 bp long cell-type-specific promoters for three relatively similar blood cancer cell lines: Jurkat, K562, and THP1. As mentioned in the introduction, designing such promoters is difficult since these cell lines are very similar compared to cell lines derived from different tissues. Moreover, Jurkat and THP1 cells are relatively understudied and lack large sources of PE measurements. We also perform experimental validation of the designed sequences to show the effectiveness of our approach.

### 5.1. Setup

#### Model training (design and ensemble models)

To train design models, we use an architecture similar to MTLucifer (Reddy et al., 2024) (shown in Figure S.1). We pretrain the models on large existing MPRA datasets before fine-tuning them using a small PE dataset consisting of 17,104 measurements collected by Reddy et al. (2024) from Jurkat, K562, and THP1 cells (dataset is described in more detail in Appendix B). We use this approach over fine-tuning Enformer, since our codebase uses Jax (Bradbury et al., 2018) for efficiently performing conservative regularization, and pretrained Enformer models are not available for Jax. Following Section 4.2, we train 5 design models, each with a different α value. We also build an ensemble for final sequence selection using a different split of the same dataset. Appendix D describes the training process in more detail.

#### Sequence design

We design 4,000 sequences for each cell type using our workflow. This number was chosen based on experimental constraints, and to keep the total number of sequences close to the size of the original training dataset. Each design model is used to design ∼17K candidate sequences (one design for every starting sequence from the fine-tuning dataset) using the process described in Section 4.2, and the final 4,000 sequences are chosen from these candidates using the algorithm from Section 4.3. As shown later in this section, the designed sequences are naturally diverse, and we set the diversity coefficient β to zero since we did not need to improve diversity.

#### Baselines

We also design sequences using two baselines, in order to determine the usefulness of various components of our workflow:

1. **Motif tiling:** This heuristic method to design cell type-specific promoters is similar in spirit to that proposed by Nissim et al. (2017), and aims to improve upon known high DE sequences. We use it to determine the overall usefulness of our approach for designing promoters compared to traditional methods. Briefly, the motif tiling approach inserts motifs associated with high DE into sequences from the fine-tuning dataset that already have high DE, with the goal of increasing their DE even further. We run it for the three target cell types to design 520, 630, and 515 sequences for Jurkat, K562, and THP1 cells, respectively. Appendix E.1 presents the details of this method.
2. **DENs:** In Section 4.2, we use gradient ascent to optimize starting sequences and produce designs that increase the design model-predicted DE. However, as detailed in the Section 2, many previous studies use generative models for this purpose. To determine which optimization algorithm is better, we replace gradient ascent with DENs in our workflow and produce 2000 sequences per cell type (with β=10). We specifically use DENs, as their designs were experimentally validated by Linder et al. (2020) and they are explicitly trained to produce diverse sequences. Appendix E.2 describes DENs in more detail.

#### Experimental validation of sequences

We use a similar protocol as Reddy et al. (2024) to experimentally determine the DE of designed sequences (Appendix C). Along with the designed sequences, we also measure the DE of the top sequences from the training dataset (top 100 sequences for each cell type, so 300 sequences total), and of ∼200 sequences from the training dataset whose expression roughly uniformly spanned the full range of PE values. These sequences with measurements in both the training set experiment and the validation experiment are used to determine whether designed sequences improve upon their starting sequences, using a procedure described later in this section. In total, we measure the DE of 20,741 sequences. After filtering out sequences that have less than 100 reads in any experiment, we are left with 14,315 high-quality measurements for use in all downstream analyses.

### 5.2. Main Results

#### Evaluating the diversity of designed sequences

We analyze the diversity of the designs in terms of their mean base pair entropy and the mean Hamming distance between any two designs (Table 1). **Overall, our approach produces highly diverse sequences for all three cell types.** We note that for THP1, DENs produce significantly less diverse designs. This is possibly due to the difficulty of the design problem - the fine-tuning dataset has few sequences with high DE in THP1, leading to the design model having fewer modes. This might make it difficult for a generative model trained from scratch to discover the design model’s modes. Since gradient ascent starts from the sequences in the fine-tuning dataset, it can more easily discover the various modes.

#### Does our approach design sequences that are better than the corresponding starting sequences?

Next, we analyze the overall effectiveness of our workflow and compare it to motif tiling. Since we aim to design promoters that have higher cell-type-specificity than promoters in the fine-tuning dataset, we need to quantify the improvement in DE from the starting sequences to the designed sequences. Direct comparison of DE values is challenging due to differences in experimental conditions and potential confounding factors like batch effects, and re-measuring the DE of all starting sequences is inefficient. To address this issue, we use a common set of sequences that span the expression range for any given cell type to calibrate DE values between experiments. Since these common set sequences have DE values from both experiments, we can compute percentile scores for both the starting and corresponding designed sequences among this common set. The design process is considered effective if the designed sequence achieves a higher percentile score than the starting sequence. This calibration is valid since the ranks of the sequences in the common set are concordant between experiments, as evidenced by the high Spearman correlation between DE values measured by Reddy et al. (2024) and our experiments: 0.953 for Jurkat, 0.921 for K562, and 0.950 for THP1.

**Figure 2:**
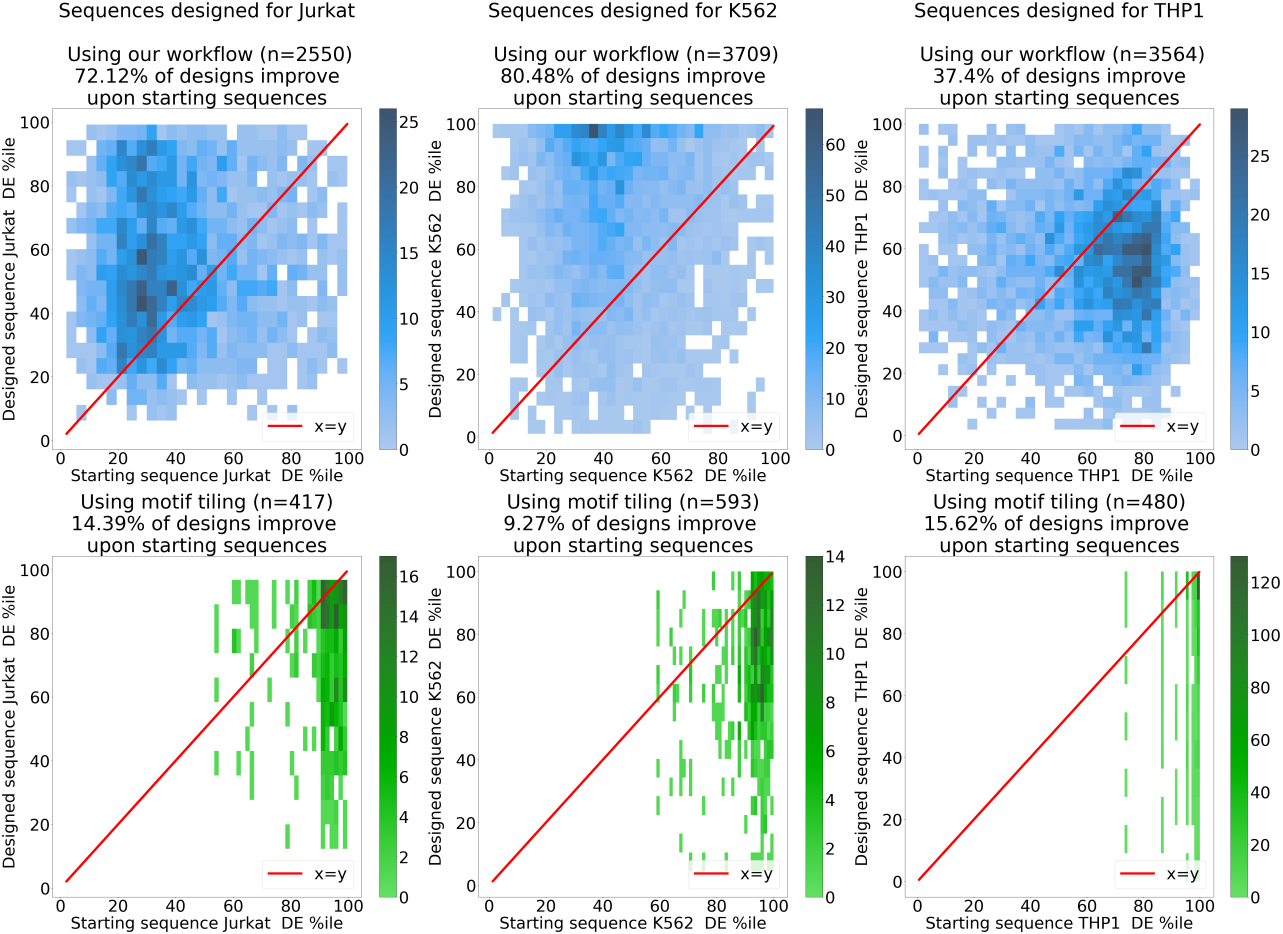
Comparing the DE of designed sequences with the DE of corresponding starting sequences. The top row of plots compare the percentile scores of the starting and designed sequences from our approach, and the bottom row of plots compare the percentile scores for starting and designed sequences from motif tiling. Each column represents sequences designed for one of the three cell types.

Figure 2 shows the percentile scores of the starting and designed sequences from our approach and from motif tiling. Sequences designed for Jurkat and K562 using our approach improve upon the majority of corresponding starting sequences, highlighting the overall effectiveness of our approach in this difficult setting. **In fact, we design a promoter for K562 that has 75.85% higher DE (=5.17) than the best high DE sequence from the fine-tuning dataset (=2.94).** On the other hand, we observe that using motif tiling to improve on high DE sequences is ineffective, almost always leading to worse sequences. We note that our workflow tends to choose designs that come from relatively mediocre starting sequences. This is possibly because most sequences in the fine-tuning dataset have DE similar to these sequences, likely making the design and ensemble models accurate and confident near such sequences. Since our workflow significantly improves upon such sequences, we should be able to discover progressively better sequences over multiple rounds of model training, design, and experimental evaluation.

However, we fail to improve upon most starting sequences for THP1. This is likely because of the difficulty of the design problem. As mentioned previously, there are few high DE sequences in the fine-tuning dataset for THP1. Thus, it might be difficult for design models to be accurate in the space of high DE sequences, leading to poor designs. In such cases, collecting more experimental data for training might be the only effective way to improve designs.

#### Gradient ascent is beEer than DENs for designing sequences

**Figure 3:**
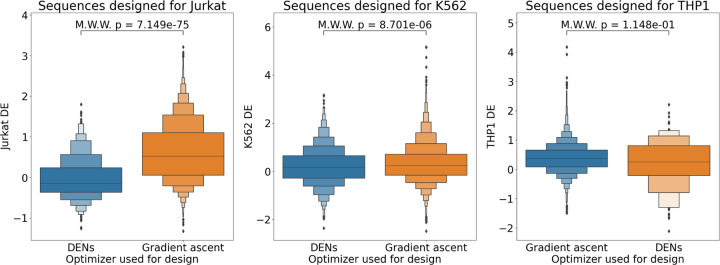
Box plots comparing the DE of sequences designed using gradient ascent as the optimizer in our workflow vs. those from DENs. p-values are computed using a Mann-Whitney-Wilcoxon (MWW) test that tests whether sequences from gradient ascent have higher DE than sequences from DENs.

Finally, we compare gradient ascent to DENs (Figure 3) to determine the better optimizer for use in our workflow. We observe that gradient ascent is generally more effective than DENs. Given that gradient ascent is also easier to implement and tune, we recommend it over generative models such as DENs.

## 6. Discussion and limitations

Designing cell-type-specific promoters is a crucial step in effective application of gene delivery technologies. We introduce a novel workflow for promoter design based on COMs (Trabucco et al., 2021), tailored for real-world applications due to its data efficiency and ability to produce diverse, non-adversarial designs. We apply this approach to the challenging task of designing cell-type-specific promoters for three similar blood cancer cell lines. We then experimentally validate our designs, demonstrating effectiveness for two out of three cell lines. We also present limitations that practitioners should consider. The success of our workflow depends on the accuracy of the design models, and effectiveness across the three cell lines was related to design model accuracy for each cell line (Table S.1). This resulted in failure for THP1 cells, where we had lower quality fine-tuning data with few high DE sequences. Pretraining data may also affect performance, and many existing MPRA datasets used for pretraining come from K562 cells, likely explaining our superior performance in K562 compared to Jurkat. Our MBO workflow highlights these and other practical considerations. Our approach is valuable for practitioners targeting a wide range of cell types, and will inspire further research in this area.

## Supplementary Material

Supplement 1

## Figures and Tables

**Table 1:** Quantifying the diversity of designs. Mean base pair entropy (maximumvalue=2) and mean pairwise Hamming distance (maximumvalue=250) are shown for the designed sequences. Mean base pair entropy is calculated by determining the entropy of each position across all designs and then averaging these values.

Method	Number of sequences			Mean base pair entropy			Mean Hamming distance		
	Jurkat	K562	THP1	Jurkat	K562	THP1	Jurkat	K562	THP1
**Our approach**	4000	4000	4000	1.87	1.96	1.96	176.18	183.73	184.17
**DENs used instead of grad. asc.**	2000	2000	2000	1.89	1.87	1.00	178.17	177.39	108.22
**Motif tiling**	520	630	515	1.79	1.90	1.92	168.71	179.37	180.95

## Footnotes

1 Designs with ensemble average predicted PE in target cell type c greater than the 90th percentile of predicted PE in c for the fine-tuning dataset’s sequences.
