# Supplementary material for: Designing Cell-Type-Specific Promoter Sequences Using Conservative Model-Based Optimization
Source: bioRxiv. 2024 Jun 23:2024.06.23.600232. Preprint. [Version 1] doi: 10.1101/2024.06.23.600232 (PMC11213138; doi:10.1101/2024.06.23.600232)
Supplement: Supplement 1 [file NIHPP2024.06.23.600232v1-supplement-1.pdf]

bioRxiv preprint doi: https://doi.org/10.1101/2024.06.23.600232; this version posted June 23, 2024. The copyright holder for this preprint (which was not certified by peer review) is the author/funder, who has granted bioRxiv a license to display the preprint in perpetuity. It is made available under aCC-BY-NC-ND 4.0 International license.

A. Supplementary Figures

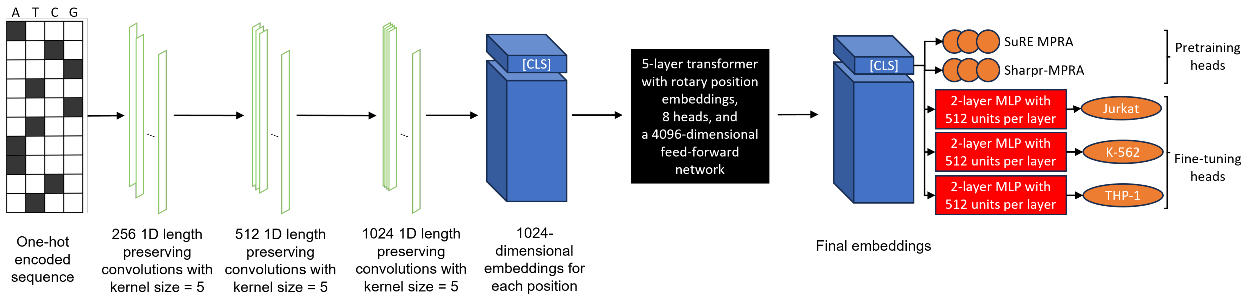

Figure S.1: Design model architecture: the convolutional layers use GELU activation (Hendrycks and Gimpel, 2016), dropout with 0.1 probability (Srivastava et al., 2014), and are followed by a group norm layer (Wu and He, 2018) with each group size being 16. The MLP layers in the fine-tuning heads also use GELU activation. We apply the RoPE position embeddings (Su et al., 2021) at each attention layer of the transformer.

B. Experimental data used for training design models

We use the PE measurements collected by Reddy et al. (2024) for designing cell type-specific promoters. They provide 17,104 PE values in Jurkat, K562, and THP1 cells derived from 250 bp long manually designed promoters. They set out to measure the PE of 20,000 promoters chosen using heuristics that try to maximize the number of differentially expressed promoters. Nearly 50% of the tested promoters are from differentially expressed endogenous genes, another $\sim 40\%$ are crafted by assembling known and de-novo motifs which are abundant in the promoters of differentially expressed endogenous genes, and the remaining $\sim 10\%$ of promoters originate from highly expressed endogenous genes' promoters. Each promoter is integrated upstream of a minimal CMV promoter and the enhanced green fluorescent protein (EGFP) reporter gene within a lentiviral vector. The resulting expression in each cell line post-transduction is quantified by the levels of induced fluorescence. They get 17,104 PE values of adequate quality with two replicates and average the values from each replicate to get the final PE values - we use these values in our experiments.

This data was released with their codebase (https://github.com/anikethjr/promoter_models) under an AGPL-3.0 license.

C. Experimental protocol for measuring promoter-driven expression of designed sequences

We used the same protocol as Reddy et al. (2024) with the following modifications for testing the newly designed sequences. First, the cycle number for PCR amplification of the Twist oligopool was reduced from 12 to 10 cycles to reduce amplification bias, and the lentiviral backbone used a CMV promoter instead of an RSV promoter to drive the expression of the cargo from the lentiviral transfer plasmid to increase titer. To generate lentivirus, we used the LV-MAX kit (ThermoFisher A35684) following the protocol for a 1L flask, followed by the same concentration method. For lentiviral titration and full scale transduction, we used the same protocol, except we did not perform spinfection as this was deemed not necessary for adequate transduction efficiency. After transduction, medium containing 8ug/mL polybrene and lentivirus was changed to fresh medium after 24 hours, and cells were allowed to recover for another 48 hours before performing puromycin selection. This additional time allowed for better expression of the puromycin resistance gene before selection, increasing functional titer. Full scale transductions were performed at >500X library coverage and at an MOI <0.3. Cells were sorted using the same general methodology at a similar coverage; however, gDNA was extracted using the NucleoSpin Blood L (Machery-Nagel 740954) with the addition of RNAse A (NEB T3018L) following the manufacturer's instructions. No changes were made to NGS library preparation, but sequencing was performed using an Illumina NovaSeq X Series, pooled with other NGS libraries with non overlapping indices.

bioRxiv preprint doi: https://doi.org/10.1101/2024.06.23.600232; this version posted June 23, 2024. The copyright holder for this preprint (which was not certified by peer review) is the author/funder, who has granted bioRxiv a license to display the preprint in perpetuity. It is made available under aCC-BY-NC-ND 4.0 International license.

D. Training of design and ensemble models and their prediction performance

In this section, we detail the process we use to train our design and ensemble models. We train 5 design models with varying values of the conservatism coefficient α ($\alpha \in \{0, 0.0003, 0.001, 0.003, 0.01, 0.03\}$). We also highlight their prediction performances.

D.1. Pretraining details

Here, we provide details about our pretraining process. All design models and all constituent models of the ensembles are pretrained before being fine-tuned. Following Reddy et al. (2024), we pretrain our models using data from Sharpr-MPRA (Ernst et al., 2016) and SuRE MPRA (van Arensbergen et al., 2017; 2019). SuRE MPRA measures the expression induced by 150-500bp genomic fragments from 4 individuals from 4 different populations in the K562 and HepG2 cell lines. $\sim$ 2.4B and $\sim$ 1.2B fragments were found to be expressed in K562 and HepG2 respectively. Most fragments have very low expression and training on all measurements is time-consuming. Thus, Reddy et al. (2024) define a classification task using this data that subsets the data and bins each sequence into one of 5 expression bins. We also use this classification task for pretraining. Sharpr-MPRA is a smaller MPRA that measures the expression from $\sim$ 487K 145bp sequences centered at DNase I peaks in K562 and HepG2 cells and in two different settings. Reddy et al. (2024) use a preprocessed version of this data from Movva et al. (2019) that builds a regression task with 12 outputs (2 replicates for expression measured in 2 settings in 2 cell lines, and 4 outputs that correspond to the average expression across replicates). We use the same formulation for pretraining our models.

During pretraining, our models' output layers produce the probability of a sequence belonging to each of the expression bins for the SuRE MPRA-based pretraining task and directly predicts the expression values for the Sharpr-MPRA-based pretraining task. We minimize the sum of the negative log-likelihood (NLL) loss for the SuRE MPRA task and the mean squared error (MSE) loss for the Sharpr-MPRA task (since both tasks have distinct sequences, a training sequence only contributes to one of the two loss terms, the other loss is set to zero for that sequence).

We use the dataset splits defined by Reddy et al. (2024) and train with a batch size of 448 using the AdamW optimizer (Loshchilov and Hutter, 2017) with 1e-4 learning rate and 3e-3 weight decay. We train the models for 20000 steps in total, with a cosine learning rate decay schedule that decays to 0. We retain the best checkpoint that is selected by monitoring the validation loss throughput the training process.

D.2. Building an ensemble by combining models with slightly different architectures

The ensemble uses constituent models that slightly modify the design model architecture presented in Figure S.1 - each model uses a different type of MLP layer (shown in red in Figure S.1) in the fine-tuning head. Within the set of constituent models, we vary the depth (2, 4, or 8 layers), number of hidden units (512, 1024, or 2048), and activation functions (tanh, GELU, ReLU, or SiLU (Hendrycks and Gimpel, 2016)) of the MLP layers. This gives us 36 constituent models for ensembling.

D.3. Fine-tuning details

After pretraining, all design models are fine-tuned using a certain split of the PE dataset collected by Reddy et al. (2024) (Split 1 in Table S.1). The ensemble's constituent models are fine-tuned using a different split (Splits 2 in Table S.1). In each split, following Reddy et al. (2024), we use $\sim$ 70% of the assayed promoters for training, $\sim$ 10% for validation, and $\sim$ 20% for testing. Since there are distinct classes of promoters in the dataset with varying levels of GC content, our splits are stratified by both promoter class and GC content.

When fine-tuning the design models using the conservative regularizer, the full fine-tuning objective from Eqn 3 is optimized for up to 200 steps using the AdamW optimizer (Loshchilov and Hutter, 2017) (batch size = 512, learning rate = 5e-5, weight decay = 3e-3, $\beta_1 = 0.9$, $\beta_2 = 0.999$) with a cosine learning rate decay schedule that decays to 0 after 10 warm-up steps and model checkpoints with the lowest validation set loss are retained.

The constituent models of the ensemble are fine-tuned without the conservative regularizer using the AdamW optimizer with a batch size of 512, 5e-5 learning rate, 3e-3 weight decay, $\beta_1 = 0.9$, $\beta_2 = 0.999$, and

bioRxiv preprint doi: https://doi.org/10.1101/2024.06.23.600232; this version posted June 23, 2024. The copyright holder for this preprint (which was not certified by peer review) is the author/funder, who has granted bioRxiv a license to display the preprint in perpetuity. It is made available under aCC-BY-NC-ND 4.0 International license.

with a cosine learning rate decay schedule that decays to 0 after 10 warm-up steps. They are fine-tuned for upto 250 steps, and we retain the best checkpoints that are selected by monitoring the validation loss throughput the training process.

D.4. Prediction performance

The prediction performance of our models on their respective test sets is shown in Table S.1. These results indicate that all of our models are accurate predictors of PE in the target cell types, justifying their usage in designing promoters.

Model	Dataset Split	Jurkat		K562		THP1	
		r	ρ	r	ρ	r	ρ
Design model with $\alpha = 0$	Split 1	0.699	0.666	0.705	0.698	0.644	0.542
Design model with $\alpha = 0.0003$	Split 1	0.700	0.666	0.706	0.698	0.644	0.542
Design model with $\alpha = 0.001$	Split 1	0.700	0.666	0.706	0.698	0.643	0.542
Design model with $\alpha = 0.003$	Split 1	0.701	0.667	0.706	0.698	0.642	0.543
Design model with $\alpha = 0.01$	Split 1	0.700	0.668	0.705	0.698	0.639	0.545
Ensemble model used for final selection	Split 2	0.702	0.659	0.691	0.690	0.627	0.529
Test Set Replicate Concordance	Split 1	0.819	0.735	0.737	0.666	0.730	0.483

Table S.1: Prediction performance obtained using our models.

E. Details about baselines

In this section, we describe the baselines in more detail.

E.1. Designing cell type-specific promoters using motif tiling

In this section, we detail how we design cell type-specific promoters using motif tiling. First, to identify motifs that might contribute to DE, we use FIMO (Grant et al., 2011) with default settings to detect instances of clustered TF-binding motifs defined by Vierstra et al. (2020) [2] in the sequences assayed by Reddy et al. (2024), and retain detected motif occurrences with q-value < 0.01. Let's now consider designing a cell type-specific promoter for Jurkat. For every motif, we first run a Welch's t-test to determine if sequences that contain it have higher expression in Jurkat than sequences that do not contain it, and retain motifs with q-values < 0.01. Then, for every motif, we run 2 pairwise Welch's t-tests to determine if its presence leads to higher expression in Jurkat compared to K562 or THP1. Motifs that have positive effect sizes in both t-tests (i.e. leads to higher expression in Jurkat compared to both K562 and THP1) with q-values < 0.01 are retained as those that could be contributing towards DE in Jurkat. Then, these motifs are used to design two sets of sequences - one set of sequences is designed by inserting the same motif into a background sequence as many times as possible while separating the motifs by 10bp (we get 5 sequences per motif using different background sequences), and the second set is designed by randomly sampling motifs (weighted by average effect size from the two pairwise tests) from the list of retained motifs and inserting as many of them as possible into a background sequence while separating the motifs by 10bp. While generating each sequence, the background sequence is a randomly chosen sequence from those assayed by Reddy et al. (2024) that exhibits a DE of at least 2 and also has PE in the target cell that is greater than the 90th percentile of PE. Inserted motif sequences are sampled from the motif's position weight matrix (PWM). The same process is repeated for K562 and THP1.

We discover 4, 26, and 3 motifs that may be causing DE in Jurkat, K562 and THP1 respectively. Thus, we get 20, 130, and 15 sequences in Jurkat, K562, and THP1 respectively by tiling the same motif repeatedly. Then, for each cell line, we design 500 sequences by randomly sampling motifs. Therefore, we get a total of 520, 630, and 515 sequences for Jurkat, K562, and THP1 respectively using this design method.

[2] https://resources.altius.org/~jvierstra/projects/motif-clustering-v2.0beta/

bioRxiv preprint doi: https://doi.org/10.1101/2024.06.23.600232; this version posted June 23, 2024. The copyright holder for this preprint (which was not certified by peer review) is the author/funder, who has granted bioRxiv a license to display the preprint in perpetuity. It is made available under aCC-BY-NC-ND 4.0 International license.

E.2. Deep Exploration Networks (DENs)

DENs are generative models that are trained to output diverse sequences that maximize a design model's predictions. The generator takes random noise as input and transforms it into a sequence PWM. We use a UNet-style (Ronneberger et al., 2015) generator that first transforms the noise vector into a sequence PWM-sized matrix (i.e. of size (250, 4)). Then, it applies 6 downsizing convolutional layers followed by 5 upsizing convolutional layers. A final convolutional layer then pools information across the final set of filters' outputs to produce the sequence PWM. The PWM is used to sample sequences that are fed to the design model to get its predictions and a fitness-based loss that trains the DEN to output high-fitness sequences is computed. Additionally, to explicitly increase the diversity of the generated sequences, in every training step, random noise vectors are input to the DEN in pairs to get two sequence PWMs per pair. Then, a diversity-based loss is computed that incentivizes the sequences generated using the two different noise vectors to be distinct from each other, both in sequence and design model embedding space. An entropy-based loss is also minimized to reduce the entropy of the PWM output by the DEN at every position.

Thus, when training a DEN to generate cell type-specific promoters for a target cell $i \in$ {Jurkat, K562, THP1}, the training objective we use is:

$$\min_{\phi} \underbrace{-\left[\sum_{\substack{j=1,\\ s_j \sim g_\phi(u_1)}}^{2} \mathrm{DE}^i_\theta(s_j) + \sum_{\substack{j=1,\\ q_j \sim g_\phi(u_2)}}^{2} \mathrm{DE}^i_\theta(q_j)\right]}_{:=\text{ fitness loss}}$$

$$+ \underbrace{\beta_{\text{diversity}} \max\left[-0.3 + \max_{\sigma\in[0,10]} \frac{1}{N-\sigma}\left[\frac{1}{2}\sum_{\substack{j=1\\ s_j\sim g_\phi(u_1)\\ q_j\sim g_\phi(u_2)}}^{2} \sum_{k=\sigma}^{N} s_{j,k}.q_{j,k-\sigma}\right], 0\right]}_{:=\text{ sequence-based diversity loss}}$$

$$+ \underbrace{\beta_{\text{diversity}} \max\left[-0.3 + \frac{1}{2}\sum_{\substack{j=1\\ s_j\sim g_\phi(u_1)\\ q_j\sim g_\phi(u_2)}}^{2} \frac{R_\theta(s_j).R_\theta(q_j)}{||R_\theta(s_j)||.||R_\theta(q_j)||}, 0\right]}_{:=\text{ embedding-based diversity loss}}$$

$$+ \underbrace{\beta_{\text{entropy}} \max\left[1.8 - \frac{1}{N}\sum_{k=1}^{N}\left[\log_2 4 - \sum -g_\phi(u_1)_k \log_2\left(g_\phi(u_1)_k + 10^{-8}\right)\right], 0\right]}_{:=\text{ entropy loss}}$$

where ϕ is the set of trainable parameters of DEN g_ϕ which outputs $N = 250$ base pairs long sequence PWMs by taking u_1 or u_2 - 200-dimensional random noise vectors sampled from the uniform distribution over [-1, 1], as inputs. From the sequence PWMs output by g_ϕ, we sample two one-hot encoded sequences per noise vector denoted by s_j and q_j. These sequences are then input to the trained design model that predicts PE induced in each of the three cell types. Then, the predicted DE in the target cell i induced by a sequence **x** is given by Eqn 1. The fitness loss maximizes this predicted DE. The other loss terms increase sequence diversity and reduce entropy in the sequence PWM. Here, $s_{j,k}$ is the one-hot encoded base pair at position k in s_j (similarly for $q_{j,k}$), $R_\theta(s_j)$ is an embedding for s_j extracted from the design model f_θ, $g_\phi(u_1)_k$ is the probability distribution over base pairs in the sequence PWM $g_\phi(u_1)$ at position k. Finally, the coefficients $\beta_{\text{diversity}}$ and β_{entropy} are used to weight the diversity and entropy losses relative to the fitness

bioRxiv preprint doi: https://doi.org/10.1101/2024.06.23.600232; this version posted June 23, 2024. The copyright holder for this preprint (which was not certified by peer review) is the author/funder, who has granted bioRxiv a license to display the preprint in perpetuity. It is made available under aCC-BY-NC-ND 4.0 International license.

loss and to one another. They can be varied to regulate the diversity vs. fitness trade-off. We refer readers to the original work by Linder et al. (2020) that proposed DENs for more details on the method. We tune the various hyperparameters reflected in the training objective by observing the overall quality of the generated sequences.

We train a total of 15 DENs per target cell type, each using a different design model to compute the fitness loss, or making a different diversity vs. fitness trade-off by using different $\beta_{\text{diversity}}$ values. We have 5 different design models, each trained using a different conservatism coefficient α (Table S.1). We also try 3 different $\beta_{\text{diversity}}$ values - 1, 5, and 10, yielding 15 DENs in total. Each DEN is used to generate 20000 sequences and we use the final sequence selection algorithm from Section 4.3 to choose the final set of 2000 sequences per target cell.
